# Solid Lipid Nanoparticles Encapsulating a Benzoxanthene Derivative in a Model of the Human Blood–Brain Barrier: Modulation of Angiogenic Parameters and Inflammation in Vascular Endothelial Growth Factor-Stimulated Angiogenesis

**DOI:** 10.3390/molecules29133103

**Published:** 2024-06-28

**Authors:** Giuliana Greco, Aleksandra Agafonova, Alessia Cosentino, Nunzio Cardullo, Vera Muccilli, Carmelo Puglia, Carmelina Daniela Anfuso, Maria Grazia Sarpietro, Gabriella Lupo

**Affiliations:** 1 Department of Biomedical and Biotechnological Sciences, School of Medicine, University of Catania, 95123 Catania, Italy; 2 Department of Chemical Sciences, University of Catania, 95125 Catania, Italy; 3 Department of Drug and Health Sciences, University of Catania, 95125 Catania, Italy; 4 NANOMED-Research Center on Nanomedicine and Pharmaceutical Nanotechnology, University of Catania, 95125 Catania, Italy

**Keywords:** SLNs, benzo[k,l]xanthene lignans, VEGF-induced angiogenesis, blood–brain barrier

## Abstract

Lignans, a class of secondary metabolites found in plants, along with their derivatives, exhibit diverse pharmacological activities, including antioxidant, antimicrobial, anti-inflammatory, and antiangiogenic ones. Angiogenesis, the formation of new blood vessels from pre-existing ones, is a crucial process for cancer growth and development. Several studies have elucidated the synergistic relationship between angiogenesis and inflammation in various inflammatory diseases, highlighting a correlation between inflammation and vascular endothelial growth factor (VEGF)-induced angiogenesis. Thus, the identification of novel molecules capable of modulating VEGF effects presents promising prospects for developing therapies aimed at stabilizing, reversing, or even arresting disease progression. Lignans often suffer from low aqueous solubility and, for their use, encapsulation in a delivery system is needed. In this research, a bioinspired benzoxantene has been encapsulated in solid lipid nanoparticles that have been characterized for their pharmacotechnical properties and their thermotropic behavior. The effects of these encapsulated nanoparticles on angiogenic parameters and inflammation in VEGF-induced angiogenesis were evaluated using human brain microvascular endothelial cells (HBMECs) as a human blood–brain barrier model.

## 1. Introduction

Lignans are a widespread secondary metabolites class found in plants. These compounds derive from a radical phenolic oxidative coupling reaction and are categorized into two main classes based on the different linkage pattern of these phenylpropane units: classical and neolignans [1,2].

They are typically present in various parts of plants, including roots, stems, bark, leaves, seeds, and fruits [3]. This heterogeneous class has demonstrated notable pharmacological activities, such as antioxidant [4,5], anti-inflammatory [6], antitumorigenic [7], antiviral [8], antimicrobial [9], cardiovascular [10], and many other various activities.

**Figure 1:**
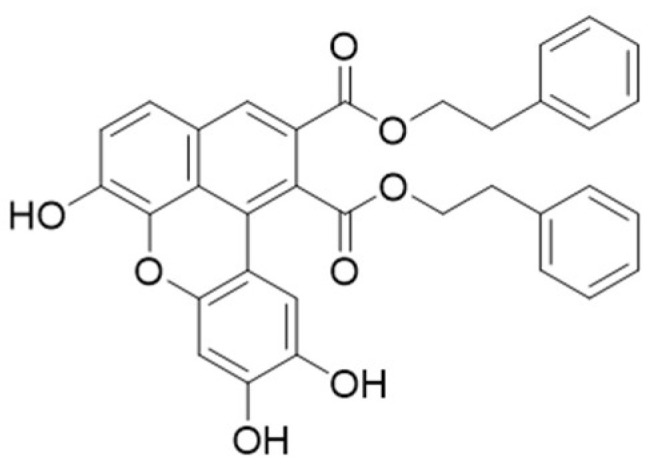
CAPE-BXL (BXL) chemical structure.

Belonging to the class of lignans, benzo[k,l]xanthenes are rarely found in nature. Tringali’s group developed a biomimetic procedure for the synthesis of bioinspired benzo[k,l]xanthenes through the oxidative coupling of caffeic esters [11]. Subsequently, synthetic derivatives were tested for various biological activities, including antiangiogenic [12], DNA-interaction [13], proteasome interaction [14], and antifungal [15]. Notably, among these synthetic derivatives the bioinspired benzo[k,l]xanthene derived from natural caffeic acid phenethyl ester (CAPE), CAPE-BXL (Figure 1), utilized in this study and henceforth referred to as BXL, has demonstrated superior antibacterial and antiproliferative activities compared to other derivatives [16]. However, its high lipophilicity (BXL: capacity factor 4.21; log *P* 6.65) and low solubility in aqueous medium make it unsuitable for pharmacological use [17]. This is one of the reasons that prompted the encapsulation of BXL into solid lipid nanoparticles (SLNs) in our study. SLNs represent an alternative colloidal carrier to traditional liposomes, emulsions, or polymeric nanoparticles and offer numerous advantages such as excellent biocompatibility, controlled and/or targeted drug release, and, ranging from 100 nm to 1000 nm, enable diverse application routes such as perioral, rectal, parenteral, ophthalmic, topical, and more [18].

As one of the most metabolically active organs, the brain relies on a dense network of microcapillaries for oxygen and nutrient supply. This network is regulated by the blood–brain barrier (BBB), a specialized system acting as both a physical and metabolic barrier between blood vessels and brain tissue [19]. Endothelial cells (ECs) play a crucial role in constituting the blood–brain barrier (BBB). Unlike those found in other tissues, these ECs form continuous tight- and adherens junctions, resulting in low paracellular flow and high transendothelial electrical resistance [20]. Astrocytes, neurons, and pericytes complete the anatomical composition of the BBB, a result of ongoing interactions among these diverse cellular components known as the “neurovascular unit” [21,22].

Angiogenesis, the formation of new capillaries from existing blood vessels, is triggered by brain tumor, which requires the formation of new vessels that can transport oxygen and nutrients for its growth. The “angiogenic switch,” marking the shift from a basal/non-angiogenic to an angiogenic state, is characterized by the activation of pro-angiogenic growth factors, such as vascular endothelial growth factor (VEGF) and fibroblast growth factor (FGF), leading to the growth of new vessels and microvessels [23].

Brain tumors, given their pronounced vascularization, emphasize the critical need for development of therapeutic strategies that specifically target angiogenesis in the tumor vasculature [24,25]. The ECs within the microvessels exhibit notable resistance to cytotoxic treatments. Additionally, these ECs display enhanced migratory capabilities and produce significant amounts of growth factors, including VEGF and factors with mitogenic properties, such as endothelin-1 (ET-1) and interleukin-8 (IL-8) [24].

Extensive evidence has demonstrated a significant correlation between VEGF-induced angiogenesis and the release of inflammatory mediators, including prostaglandins (PGE2) [26,27,28]. PGE2 contribute to angiogenesis, promoting the growth and progression of tumors. The effects of PGE2 are mediated through its binding to specific receptors, EP2 and EP4, implicated in endothelial migration/tubulogenesis [29] tumor proliferation and invasion [30].

Our previous findings showed the existence of an autocrine COX-2-prostaglandin-VEGF axis in an in vitro angiogenic model [31,32] and in a triple cell culture system representing an in vitro blood–brain barrier affected by glioma [29].

VEGF plays a significant role in the upregulation of chemokines, a superfamily of cytokine-like proteins. Notably, both VEGF and Interleukin-8 (IL-8), a pro-inflammatory chemokine, are secreted by ECs, thereby promoting migration and angiogenesis. The heightened levels of IL-8 are particularly noteworthy, as they play a crucial role in regulating endothelial permeability during the developmental processes of vascular diseases [33]. Moreover, the overexpression of IL-8 has been associated with pivotal aspects of tumor progression, including growth, metastasis, chemoresistance, and angiogenesis [34].

Hence, investigating therapeutic strategies that concurrently target both VEGF and VEGF-mediated molecular pathways is crucial for disrupting tumor-like angiogenesis and mitigating the inflammatory microenvironment. In this study, we encapsulated BXL, known for its diverse pharmacological activities, into SLNs, thoroughly characterized for their pharmaco-technological and biological parameters. This approach holds potential for assessing the efficacy of BXL in disrupting angiogenesis and attenuating the inflammatory microenvironment.

In this study, the effect of encapsulated BXL into SLNs on human brain microvascular endothelial cells (HBMECs) was investigated in terms of the modulation of cell proliferation and migration, tubulogenesis, inflammatory PGE2 production, and IL-8 release induced by VEGF.

## 2. Results and Discussion

### 2.1. SLNs Characterization

**Figure 2:**
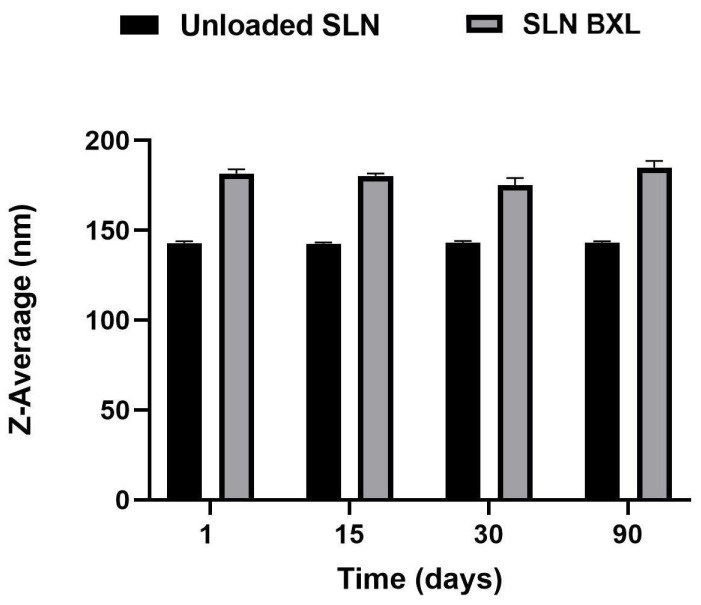
SLNs mean particle size. The results are shown as means ± SD.

**Figure 3:**
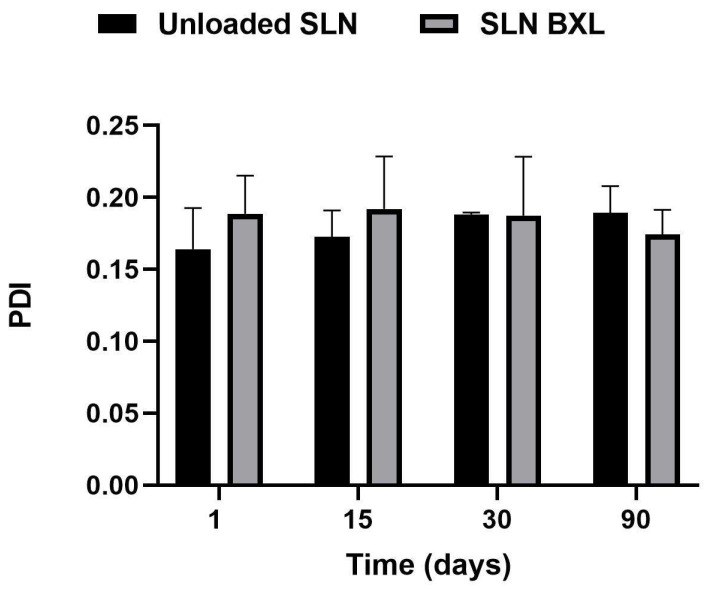
SLNs polydispersity index (PDI). The results are shown as means ± SD.

**Figure 4:**
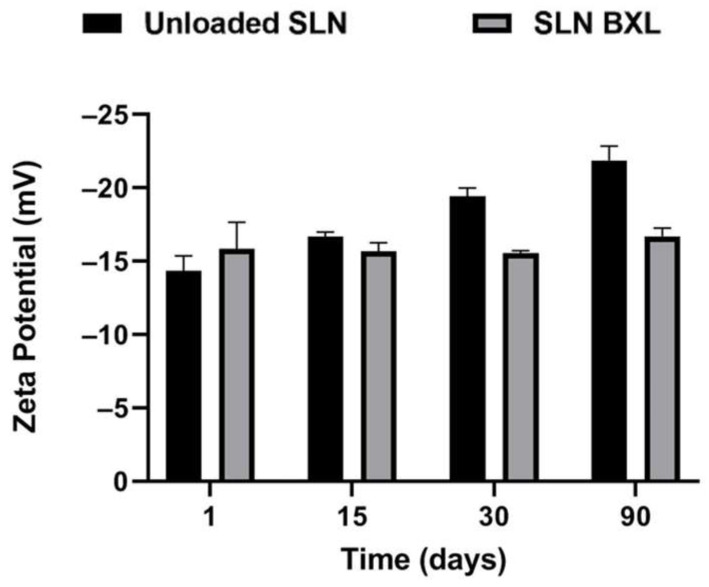
SLNs zeta potential. The results are shown as means ± SD.

Researchers have focused on SLNs as a versatile drug delivery system capable of enhancing the solubility profile of hydrophobic molecules [35]. In this study, SLNs were formulated using the Phase Inversion Temperature combined with the Ultrasonication method. To assess the stability of these formulations, it was necessary to monitor three key parameters over time: mean particle size, polydispersity index (PDI), and zeta potential. Maintaining the particle size of SLNs within a narrow range is crucial to prevent particle agglomeration during storage. Small particle sizes, particularly below 300 nm, have been reported to enhance drug bioavailability and cellular uptake. The PDI serves as an indicator of particle size distribution width, with low PDI values (<0.3) signifying a narrow size distribution and values exceeding 0.5 suggesting low homogeneity and potential particle aggregates [36]. Additionally, the zeta potential, representing the surface charge of nanoparticles, is strongly correlated with their stability. In this study, these parameters were analyzed over a period of three months at 25 °C. The mean particle size of unloaded SLNs was measured at 142.8 nm, with a PDI of 0.164. Encapsulation of the active compound led to an increase in mean particle size to 181.6 nm with a PDI of 0.189, both of which remained stable throughout the three-month period. The zeta potential of unloaded SLNs exhibited a gradual decrease, stabilizing at approximately −22 mV on the ninetieth day, while SLN-BXL maintained a zeta potential of approximately −18 mV, indicating stability over the monitoring period (Figure 2, Figure 3 and Figure 4). The observed nanoparticle size below 200 nm aligns with acceptability for nanomedical applications [37], while a PDI below 0.2 indicates a homogeneous system. The surface charge of nanoparticles, exceeding the threshold values for agglomeration (±15 mV), supports their dispersion and confers physical stability to the preparation [38]. SEM images of the SLNs and SLN-BXL showed a uniform and spherical shape of the particles, with average dimensions in agreement with the reported values (Figure S1).

### 2.2. Entrapment Efficiency and Drug Loading

The entrapment efficiency (EE%) of SLN-BXL was assessed using quantitative ^1^H NMR analysis, a technique that has gained prominence in metabolomics for its suitability in quantitatively measuring multicomponents within complex mixtures, such as natural-product isolates and drug formulations [39]. Notably, quantitative NMR has been reported to have a quantitative inaccuracy of less than 2.0%, meeting the acceptable limit for accurate quantification.

As outlined in the experimental section, both loaded and free BXL can be determined by means of a purification step using Sephadex-LH20 liquid chromatography. Precisely the elution of Sephadex-LH20 with water furnish a fraction containing SLN-BXL, whereas the elution with acetone gives a fraction containing free CAPE-BXL. ^1^H-qNMR analysis of the two fractions allows direct (aqueous fraction) and indirect (acetone fraction) quantification of EE% [40]. According to the data obtained, BXL was loaded onto SLNs with a satisfactory EE% ranging from 57.6 to 65.3%. An average EE% value of 61.5% was considered for further studies, with a drug loading of 0.031% ± 0.001, as determined by Equation (3).

The CAPE-BXL-SLNs were stored at room temperature for three months, and the EE% was monitored at time intervals. The EE% was calculated as 60.7, 60.1, and 58.2%, respectively, at 1, 2, and 3 months, thus indicating a good entrapment efficiency and stability of CAPE-BXL into the SLNs for the time period studied.

### 2.3. Release of BXL from SLNs

The in vitro release of BXL from SLNs was carried out by employing the dialysis method in 50 mM TRIS buffer at pH 7.4. Samples were collected hour by hour up to 8 h, and then, after 24 h. The BXL was quantified by ^1^H NMR. The BXL was retained into the SLNs for the time period studied.

### 2.4. Differential Scanning Calorimetry

#### 2.4.1. SLNs and SLN-BXL Calorimetric Analysis

**Figure 5:**
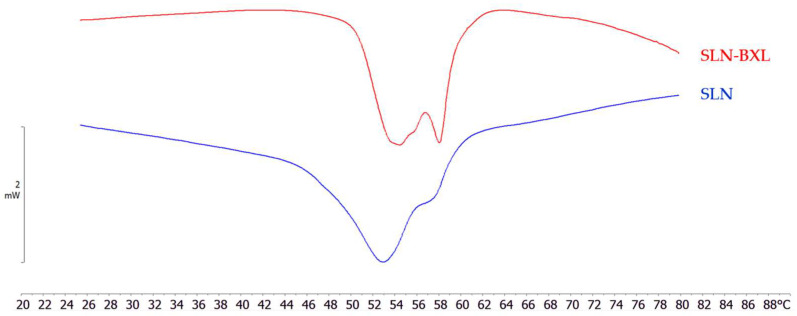
Calorimetric curves, in heating mode, of unloaded SLNs and SLN-BXL.

The thermodynamic characterization of a system allows making various assessments about the interactions among different components of the system. The DSC records the temperature-dependent heat capacity of a sample submitted to heating and/or cooling scans. From this measure, several data of thermodynamic parameters are obtained, among which the transition temperature (T_m_) is the most important. The DSC analysis is required for the study of the thermotropic behavior of the nanoparticles, and the comparison of unloaded and BXL-loaded SLNs can provide important information (Figure 5). The calorimetric curve of unloaded SLNs is characterized by a main peak at 53 °C and a shoulder at ~56 °C. In the SLN-BXL curve, it is possible to distinguish two peaks, the first at 54.3 °C and the second at 58 °C, and in the middle between these two peaks a shoulder, which began at 55.3 °C. This important difference in the conformation of the calorimetric curves indicated that BXL was encapsulated into the SLNs. The peak at higher temperatures could imply that BXL is localized in the SLNs core, interacting with the lipid molecules and, hence, affecting their thermotropic behavior; the event modified the thermotropic behavior of the nanoparticles.

#### 2.4.2. MLV-SLNs Interaction Analysis: Kinetics Studies

**Figure 6:**
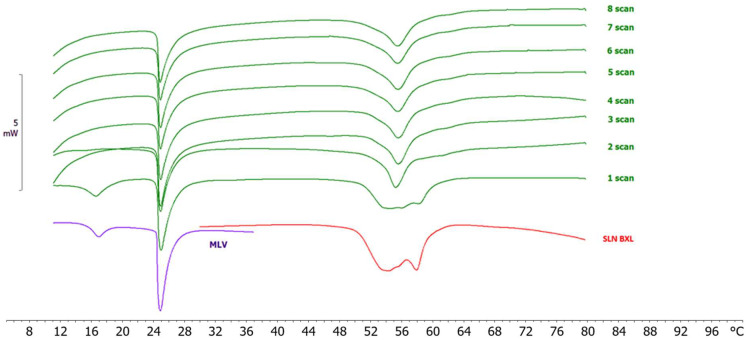
Calorimetric curves, in heating mode, of SLN-BXL and MLVs placed in contact at increasing incubation time.

Kinetic studies were conducted to assess potential interactions between biomembranes and SLNs. Multilamellar vesicles (MLVs) composed of 1,2-dimyristoyl-sn-glycero-3-phosphatidylcholine (DMPC) were employed as a simplified system mimicking biomembrane properties. Upon heating, the phospholipid bilayers within MLVs undergo transitions from an ordered (gel) phase to a ripple phase, and subsequently to a disordered (liquid–crystalline) phase, characterized by precise transition temperatures (T_m_) and enthalpy variations (ΔH). Interaction with foreign compounds can induce variations in T_m_ and ΔH. MLVs were exposed to nanoparticles, and calorimetric curves were recorded at regular intervals (Figure 6). The MLV curve, consistent with literature data [41], exhibited a pre-transition peak around 17 °C, associated with the gel-to-ripple phase transition, and a main peak at 25 °C, corresponding to the transition from the ripple phase to the disordered liquid crystalline phase of DMPC.

Contact between loaded SLNs and MLVs indeed altered the thermotropic behavior of both, as evidenced by changes in the calorimetric curves. From the second scan onwards, the pre-transition peak in the MLV curve disappeared, and the enthalpy of the main peak was reduced, while the multi-peak pattern of SLN-BXL was lost. In subsequent scans, the SLN-BXL peak resembled that of unloaded SLNs. These variations in the calorimetric curves of MLVs and SLNs suggest their interaction, and the transition of the loaded SLNs curve towards that of unloaded.

### 2.5. Cell Viability

**Figure 7:**
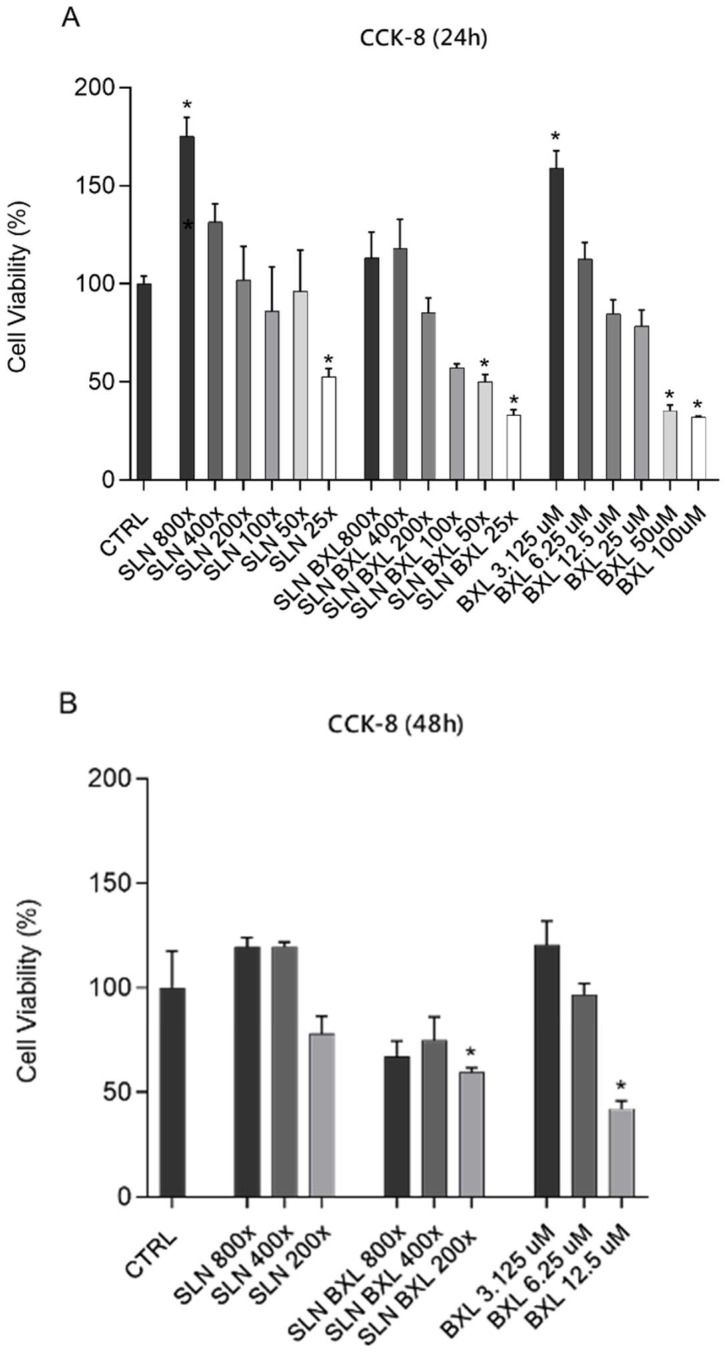
(**A**) Cell viability determined by CCK-8 assay of HBMECs grown in basal culture medium (control: CTRL) and in culture medium containing SLNs alone at 800×, 400×, 200×, 100×, 50×, 25× dilutions, or SLN-BXL at 800×, 400×, 200×, 100×, 50×, 25× dilutions (corresponding, respectively, to 3.125 μM, 6.25 μM, 12.5 μM, 25 μM, 100 μM BXL for 24 h), or BXL alone at 3.125 μM, 6.25 μM, 12.5 μM, 25 μM, 100 μM concentrations. (**B**) Cell viability evaluated at 48 h: due to the excessive toxicity inflicted by the higher concentrations, the incubations were performed only at 800×, 400×, 200× dilutions for SLNs alone, or SLN-BXL at 800×, 400×, 200× dilutions (corresponding, respectively, to 3.125 μM, 6.25 μM, 12.5 μM for BXL), or BXL at 3.125 μM, 6.25 μM, 12.5 μM concentrations). The IC_50_ value was 29.48 μM (non-linear regression for the calculation of the dose-response curve). The bars represent means ± SD of three independent experiments performed in triplicate. Statistically significant differences determined by one-way ANOVA followed by Dunnett’s multiple comparisons test are indicated as follows: * *p* ≤ 0.05 CTRL vs. treatments.

To evaluate the impact on cell viability of SLNs, SLN-BXL, and BXL, time and concentration dependent, HBMEC responses have been assayed by CCK-8 viability test (Figure 7). HBMECs were exposed to various concentrations of SLNs (800×, 400×, 200×, 100×, 50×, 25×), SLN-BXL (at equivalent dilution ratios of SLNs representing free BXL concentrations), and free BXL (ranging from 3.125 to 100 μM) for 24 h (Figure 7A). Each sample was tested in triplicate.

Both SLNs and SLN-BXL at concentrations of 800× and 400×, as well as 3.125 μM and 6.25 μM, did not affect cell viability. However, at 200× concentration, SLNs showed no cytotoxic effects, whereas both SLN-BXL at 200× concentration and 12.5 μM BXL slightly reduced viability by approximately 15%. In contrast, BXL alone induced significant toxicity, with reductions in viability reaching 65% and 68% at concentrations of 50 μM and 100 μM, respectively. Due to the substantial reduction in cell viability induced by SLN-BXL-loaded nanoparticles—43%, 50%, and 77% at 100×, 50×, and 25× concentrations, respectively—these concentrations were not tested in the 48 h viability experiments (Figure 7B). At 48 h, concentrations of 800× and 400× SLNs, as well as 3.125 μM and 6.25 μM BXL, did not affect cell viability. However, concentrations of 200× and 12.5 μM induced cytotoxicity of nearly 22% and 45%, respectively. SLN-BXL also caused reductions in viability of 19%, 25%, and approximately 40% at 800×, 400×, and 200× dilutions, respectively.

The different proliferative response of HBMECs to low and high doses of SNL, BXL, and SLN-BXL could be interpreted as a hormetic response of the cells [42,43,44].

After careful evaluation, the 200× dilution for both SLNs and SLN-BXL, corresponding to a BXL concentration of 12.5 μM, was selected for subsequent experiments.

### 2.6. Wound Healing Assay

A fundamental event accompanying the process of neo-angiogenesis is the migration of endothelial cells: driven by a gradient of cytokines and growth factors, the pre-existing endothelium proliferates and migrates towards the site of angiogenesis, providing a source of tip cells that guides the growth of a new vascular system [45]. In this context, endothelial migration necessitates the degradation of the existing extracellular matrix and basement membrane surrounding quiescent endothelial cells, enabling them to migrate and colonize new spaces [46,47]. Pathological angiogenesis is characterized by the uncontrolled motility of endothelial cells stimulated by increased concentrations of VEGF-A [48]. VEGF is the primary growth factor responsible for endothelial activation, simultaneously participating in the activation of proinflammatory cells, such as circulating macrophages and T cells, and inducing local production of metalloproteinases (MMPs), thereby promoting endothelial cell migration [46,49,50]. For this reason, the in vitro wound healing test was performed, stimulating the pro-angiogenic condition that occurs in pathological contexts.

**Figure 8:**
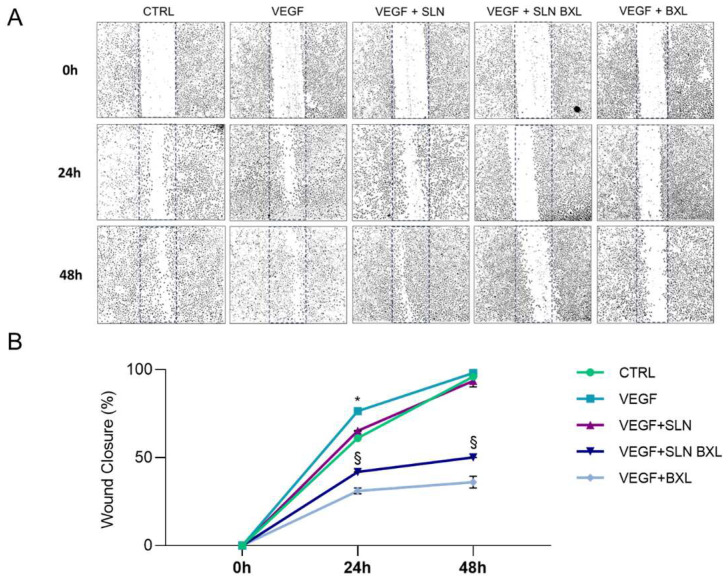
Representative micrographs (**A**) and quantitative analysis of cell migration (**B**) of HBMECs incubated with SLNs or SLN-BXL at 200× dilution, or BXL at corresponding concentration of 12.5 μM, in the absence (negative control, CTRL) or presence of VEGF-A (40 ng/mL), at 0 h, 24 h, and 48 h post-wound. Cell migration was calculated as a percentage of wound closure at each time point (0 h, 24 h, and 48 h), and expressed as means ± SD from three independent experiments. Statistical analysis was performed by two-way ANOVA, followed by Dunnett’s multiple comparison test. * *p* ≤ 0.05 VEGF vs. CTRL; § *p* ≤ 0.05 VEGF + SLNs and VEGF + BXL vs. VEGF + SLN-BXL.

Consequently, the effects of SLNs, SLN-BXL (both at 200× concentration), and 12.5 μM BXL on HBMEC motility stimulated by VEGF-A were assessed through wound healing assays (Figure 8). As anticipated, VEGF-A promoted cell migration compared to untreated control cells, leading to a significant increase in wound closure by 1.25-fold at 24 h. By 48 h post-wounding, the coverage of the scratched areas was similar in controls and VEGF-stimulated HBMECs. In the presence of the growth factor, SLNs alone did not affect cell migration at either 24 or 48 h of incubation. SLN-BXL, however, induced significant inhibition of cell migration, reducing it by 44% and 49% at 24 and 48 h of incubation, respectively. This demonstrates that BXL, known for its antiangiogenic capability [51], retained this property, even when delivered via SLNs. BXL reduced wound closure by 60% and 63% at 24 and 48 h, respectively.

### 2.7. Tube Formation

**Figure 9:**
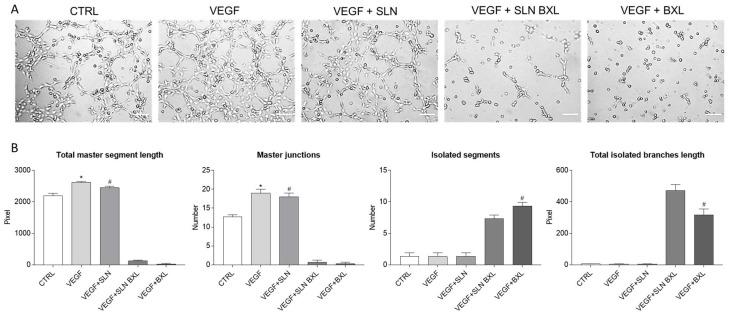
Evaluation of the angiogenic potential of VEGF-stimulated HBMECs after 6h incubation with SLNs, SLN-BXL, and BXL. (**A**) Representative microphotographs showing three-dimensional cultures in Matrigel of each sample. Magnification: 100×. Scale bar: 50 μm. (**B**) Quantification of the main parameters describing tube formation: total master segments length; number of master junctions; number of isolated segments; total isolated branches length. Values are expressed as means ± SD of three independent experiments (n = 3). Statistical analysis was performed by one-way ANOVA, followed by Tukey’s multiple comparisons test. * *p* < 0.05 VEGF vs. CTRL; # *p* < 0.05 VEGF + SLNs and VEGF + BXL vs. VEGF + SLN-BXL.

In vitro 3D tube formation assay was also performed to understand whether SLN-BXL was able to modulate VEGF-induced angiogenesis in HBMECs (Figure 9). When plated on Matrigel matrices, endothelial cells form capillary-like structures within a few hours, and this process is further enhanced by the VEGF-A treatment, as shown by the phase contrast micrographs of the tubular organization of HBMECs in Matrigel (Figure 9A). To quantify the angiogenic sprouting, some key parameters of the capillary-like patterns were considered to describe the effect of SLNs, SLN-BXL, and BXL [52] (Figure 9B). As expected, the total master segment lengths (sum of the length of the detected master segments) and the number of master junctions (angiogenic sprouting indicators) significantly increased by 1.35% and almost 1.6-fold, respectively, when cells were stimulated by VEGF; in an opposite way, no capillary-like organization was observed for either SLN-BXL or BXL, highlighting a significant in vitro anti-angiogenic effect of either SLN-BXL or BXL. In this regard, the elevated number of isolated segments (5- and 6-fold higher in cells treated with SLN-BXL and BXL, respectively, in the presence of VEGF) and the significant presence of total isolated branch length were observed, inversely correlating with the previous two parameters, confirmed the absence of tube formation when HBMECs were incubated with either SLN-BXL or BXL in medium supplemented with VEGF. Therefore, the quantitative evaluation of tubulogenesis demonstrated the anti-angiogenic effect of the BXL-loaded nanoparticles, which confirms its high encapsulation efficiency. As for SLNs alone, no inhibition of VEGF-induced vascularization was observed.

### 2.8. PGE2 Secretion in HBMEC Media

Among the angiogenic factors, eicosanoids derived from cyclooxygenase-2 (COX-2) play a prominent role, including prostaglandin E2 (PGE2). These molecules directly stimulate the synthesis of angiogenic factors, promote endothelial migration and tube formation, and support endothelial survival and migration [53]. It has been reported that mice lacking the EP2 eicosanoid receptor produce significantly less vascularized tumors than wild-type mice [54]. Moreover, the EP2 receptor has been shown to be directly involved in endothelial cell migration [55]. Our previous data from in vitro models of brain tumor and human retinoblastoma triple culture models have shown that COX-2 activation and PGE2 production in endothelial cells stimulated by tumor cells were coincident phenomena [27,56]. After 24 h of incubation, the release of PGE2 into the medium by VEGF-A-stimulated HBMECs increased by almost 1.8-fold, aligning with previous findings demonstrating the close relationship between PGE2 and VEGF in angiogenesis in human vascular endothelial cells [57]. Moreover, a kind of feed-forward loop between the phospholipid hydrolyzing phospholipase A2-COX-2-prostaglandin axis and VEGF appears to operate in the in vitro ocular angiogenic model [31,32]. Unlike BXL, which did not cause any modulation in PGE2 secretion in VEGF-stimulated cells, BXL-SLNs significantly reduced the PGE2 secretion by almost 47%, indicating a strong modulation of the intracellular enzymatic activities in HBMECs, aimed at modulating the metabolism of arachidonic acids, precursors of eicosanoids. SLNs alone did not affect the secretion of PGE2.

**Figure 10:**
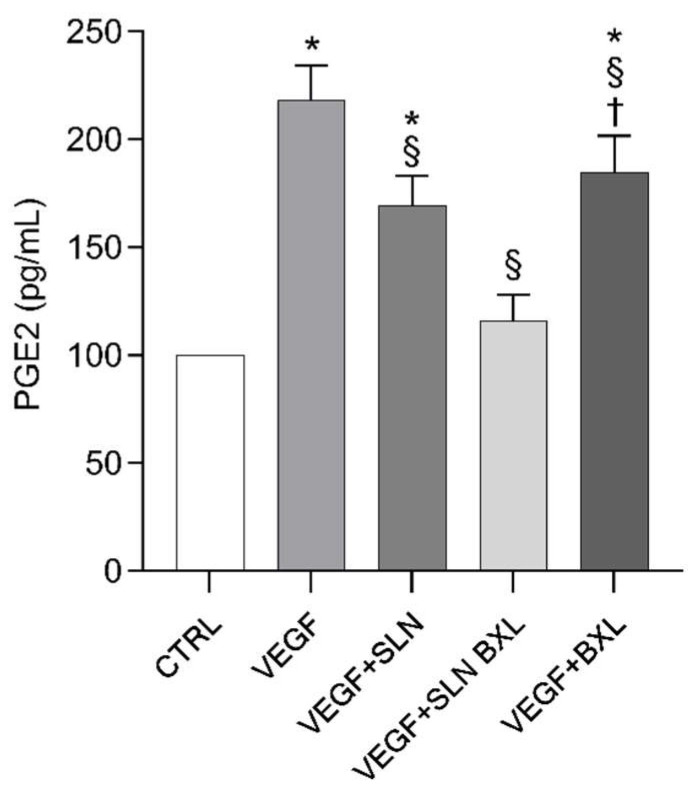
PGE2 levels in the HBMEC supernatants from control (CTRL), VEGF-A, and VEGF-A in co-incubation with SLNs, SLN-BXL, or BXL, measured by the ELISA method. Values are expressed as means ± SD of three independent experiments (n = 3). Statistical analysis was performed by two-way ANOVA, followed by Dunnett’s multiple comparison test. * *p* < 0.05 vs. CTRL; § *p* < 0.05 vs. VEGF-A; † *p* < 0.05 vs. SLN-BXL + VEGF-A.

Figure 10 shows the PGE2 levels from culture media in VEGF-A-stimulated HBMECs in the presence of SLNs, SLN-BXL, or BXL alone. Confirming previous data demonstrating a direct correlation between VEGF and PGE2 [56], here we showed that VEGF-A significantly stimulated PGE2 production by approximately 2.2-fold compared to control cells, indicating, once again, the close correlation between the pro-angiogenic stimulus inflicted by the growth factor and the cellular inflammatory reaction. Compared to a reduction in eicosanoid production of 15% induced by BXL alone, BXL-loaded SLNs caused the inhibition of PGE2 synthesis and secretion in supernatants by approximately 50%, indicating a significant inhibition of the inflammatory process, which is necessary for neo-angiogenesis and emphasizes aberrant angiogenesis [29], such as that which occurs and characterizes cerebral pathological events mediated by VEGF, including tumors, hypoxia, and stroke [58,59]. Unlike BXL alone, BXL-loaded SLNs actively affect the inflammatory response of endothelial cells following incubation with the growth factor. We hypothesize that SLNs penetrating inside HBMECs induced an intracellular pathway that negatively affected the production of PGE2 evoked by VEGF. These data are consistent with previous experiments demonstrating the ability of CAPE to inhibit the NF-kB pathway [60], lipoxygenase activity [61], and protein tyrosine kinases [62]. Additionally, CAPE has been shown to reduce vascular permeability and VEGF production after brain injury in Sprague–Dawley rats [63].

Moreover, CAPE suppressed VEGF-induced proliferation, migration, and tube formation, and decreased VE-cadherin in HUVECs, indicating the inhibition of VEGF receptor-2 and the activation of its downstream signals [64].

### 2.9. IL-8 Secretion in HBMEC Media

**Figure 11:**
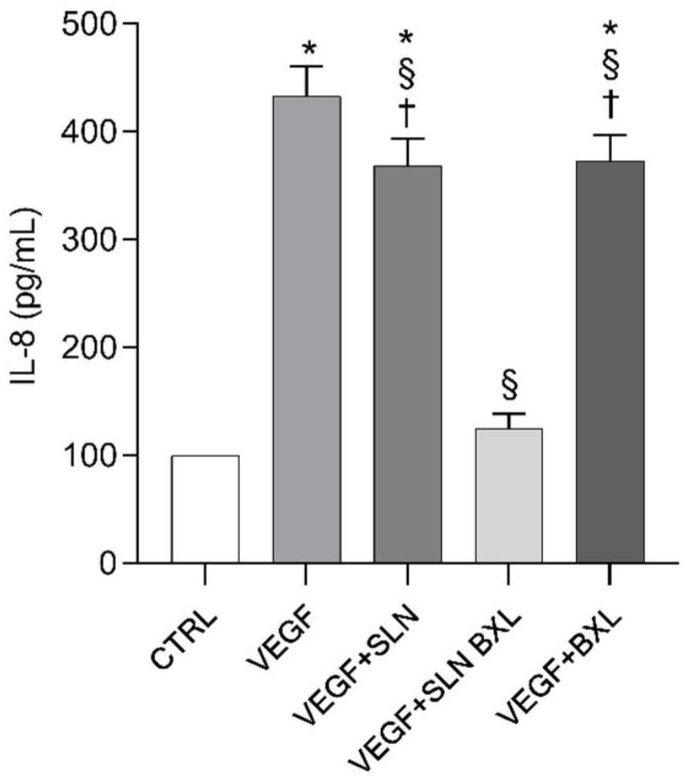
IL-8 levels in the HBMEC supernatants from control (CTRL), VEGF-A, and VEGF-A in co-incubation with SLNs, SLN-BXL, or BXL, measured by the ELISA method. Values are expressed as means ± SD of three independent experiments (n = 3). Statistical analysis was performed by two-way ANOVA, followed by Dunnett’s multiple comparison test. * *p* < 0.05 vs. CTRL; § *p* < 0.05 vs. VEGF-A; † *p* < 0.05 vs. SLN-BXL + VEGF-A.

As expected, consistent with previous findings [65,66], VEGF-A induced an almost 4.35-fold increase in IL-8 secretion in HBMECs compared to the control (Figure 11). Neither SLNs nor BXL alone exerted any significant effect on the production of this inflammatory cytokine. However, co-incubation of the growth factor with SLN-BXL led to a notable decrease in IL-8 levels by approximately 71%, suggesting a significant protective effect of BXL associated with nanoparticles on the inflammatory response of HBMECs to endothelial growth factor.

Notably, the response of VEGF-A-stimulated cells differed significantly in the presence of BXL alone compared to BXL loaded onto SLNs: only the SLN-BXL system successfully modulated the inflammatory response in endothelial cells exposed to the growth factor. This could be attributed to a more effective interaction, mediated by SLNs, with membrane receptors or facilitated the entry of BXL inside the cells, potentially activating signaling events that modulate their response to VEGF-A. Currently, the molecular mechanisms underlying the inhibitory effect of SLN-BXL on VEGF-mediated angiogenesis, and the secretion of PGE2 and IL-8, remain unknown, and further experiments are warranted to elucidate the intracellular pathways evoked by the association of BXL with SLNs in triggering the cellular response.

It can be speculated that the association of SLN-BXL induces an antiangiogenic/anti-inflammatory response in endothelial cells through negative modulation of the VEGF receptor. In line with this, a study demonstrated that BXL suppressed VEGF-induced proliferation, tube formation, migration, and loss of VE-cadherin at adherent junctions in endothelial cells by inhibiting VEGF-mediated VEGF receptor-2 activation and downstream signal activation [64].

Previously, we showed that VEGF induced the phosphorylation of PKC-α, ERK1/2, and cytosolic calcium-dependent PLA2 via the PI3-K/PKD1 pathway. Production of prostaglandins (mainly PGE2) and thromboxanes from arachidonic acid, liberated by the rapid activation of Ca2+-independent PLA2 and translocation to membranes of cPLA2, induced brain microvascular endothelial cell proliferation, migration, and vascular permeability in vitro. Both MAP kinase and cPLA2 activation were mediated by the PI3-K/PDK1 pathway [67]. VEGF is known to be involved in intracellular signaling mediated by ERK, PI3-kinase, protein kinase C, calcium, and NF-kB in endothelial cells [66], and this last transcription factor has been shown to induce IL-8 expression in endothelial cells upon treatment with various stimuli [68,69]. For all these reasons, we hypothesize that an interaction between BXL-loaded SLNs (rather than BXL alone) with VEGF-targeted or VEGF-related receptor systems may modulate the inflammatory and anti-angiogenic response stimulated by the growth factor.

## 3. Materials and Methods

### 3.1. Materials

Precirol^®^ ATO 5 (glyceryl distearate) and Gelucire 50/13, which is a mixture of mono, di-, and triglycerides and PEG-32 (MW 1500) mono- and diesters of palmitic (C16) and stearic (C18) acids, were a generous gift from Gattefossé (Saint-Pries, France). Tween^®^ 80 (polysorbate 80) was purchased from Sigma-Aldrich Co. (St. Louis, MO, USA). The chemical 1,2-dimyristoyl-sn-glycero-3-phosphocholine (DMPC) was acquired from Genzyme (Liestal, Switzerland). Purified water from Millipore-Q^®^ Gradient A10TM ultra-pure water system (Millipore, Guyancourt, France) was produced as needed and employed for the duration of the studies.

The BXL employed in this study was obtained starting from the naturally occurring CAPE (caffeic acid phenethyl ester), according to the methodology previously reported [15]. The purity of compounds was verified by ^1^H NMR and HPLC-UV (93%).

Human brain microvascular endothelial cells (HBMECs), endothelial cell medium (ECM), fetal bovine serum (FBS), and penicillin/streptomycin (P/S) solution were purchased from Innoprot (Derio, Bizkaia, Spain). Cell Counting Kit-8 (CCK-8), for quantitation of viable cell number for proliferation and cytotoxicity analysis, and Corning^®^ Matrigel^®^ Basement Membrane Matrix, for the tube formation experiments, were from Sigma-Aldrich. Recombinant human vascular endothelial growth factor A (VEGF-A) and ELISA kits were from R&D systems^®^ (Minneapolis, MN, USA).

### 3.2. SLNs Preparation

The method employed for preparing both loaded and unloaded solid lipid nanoparticles (SLNs) involved a combination of phase inversion temperature (PIT) and ultrasonication techniques. Initially, two distinct phases were prepared: the lipid phase, comprising 500 mg of Precirol^®^ ATO 5 (with a melting point of 55 °C) and 200 mg of Gelucire 50/13, and the aqueous phase, consisting of 20 mL of MilliQ water. Both phases were heated to 75 °C, approximately 20 °C above the melting point of the primary lipid. Subsequently, the aqueous phase was added dropwise to the lipid phase under magnetic stirring (300–400 rpm), yielding a pre-emulsion. This pre-emulsion was further subjected to ultrasonication using UP400S (Ultra-Schallprozessor, Dr. Hielscher GmbH, Teltow, Germany) for 6 min at an amplitude of 50%. Upon completion, the formulation was cooled to room temperature, supplemented with Tween-80 (1% *w*/*w*, 190 μL), and adjusted to a volume of 20 mL under magnetic stirring for an additional 6 min. For SLNs loaded with BXL (SLN-BXL), the active compound (10 mg), dissolved in DCM, was introduced into the lipid phase at the outset of the preparation, allowing DCM to evaporate during the heating process.

### 3.3. SLNs Characterization

#### 3.3.1. Particles Size, Polidispersity Index, and Zeta-Potential

The mean particles size (Z-average) and polidispersity index (PDI) of SLNs were measured by dynamic light scattering (DLS) method using a Zetasizer Nano-ZS90 (Malvern Instrument Ltd., Worcs, UK), equipped with a solid-state laser, with a nominal power of 4.5 mW with a maximum output of 5 mW at 670 nm. The scattering angle was 90° and the temperature was 25 ± 0.2 °C. Zeta-potential (ζ) was determined by electrophoretic light scattering (ELS) technique. For both analyses, the samples (SLN-BXL and unloaded SLNs) were diluted 1:10 (% *v*/*v*) using MilliQ water.

#### 3.3.2. Determination of the encapsulation efficiency and drug loading

CAPE-BXL-SLNs (500 µL of formulation, corresponding to a content of 0.25 mg of BXL) were subjected to size-exclusion liquid chromatography on a Sephadex-LH20 (1.0 × 10 cm) eluted with water (15 mL), with 96% EtOH (10 mL) and with acetone (10 mL). In these conditions, SLNs containing CAPE-BXL eluted in the water fraction, whereas free CAPE-BXL eluted in the acetone fraction [40]. The fractions collected were dried under vacuum and then subjected to quantitative ^1^H NMR analysis to determine the entrapment efficiency [70]. The spectra were run on a Varian Unity Inova spectrometer (Milan, Italy) operating at 499.86 (^1^H) at 300 K. Chemical shifts (δ) are indirectly referred to TMS using residual solvent signals (δ 2.05 for acetone-*d*_6_). For quantitative purposes, the area of the singlet at 6.71 ppm, attributed to the H-8 of the benzoxanthene core of CAPE-BXL, was used. A pure sample of CAPE-BXL was employed as an external standard to acquire the ^1^H NMR spectrum at different concentrations (0.12–0.350 mg/mL) to obtain the calibration curve of integrated signal vs. CAPE-BXL concentration (y = 6.95x + 0.16, R^2^ = 0.9954). Linear regression analysis allowed the estimation of CAPE-BXL (mg/mL) as BXL_entrapped_ from the aqueous fraction and as BXL_free_ from the acetone fraction. From this data it is possible to obtain the amount (in mg) of benzoxanthene relating to the two fractions, and finally, the entrapment efficiency % (EE%) was calculated indirectly and directly according to Equations (1) and (2), respectively:

(1) $$EE\%={(mgBXL}_{entrapped}÷{mgBXL}_{tot})\times 100$$

(2) $$EE\%={[(mgBXL}_{tot}-{mgBXL}_{free})÷{mgBXL}_{tot}]\times 100\;$$

From Equation (1) the EE% was 57.6% ± 4.3, from Equation (2) 65.3% ± 2.8.

Drug loading was calculated from Equation (3)

(3) $$[DL\%={(weight\;BXL}_{tot}-{weight\;BXL}_{free})÷weight\;SLN]\times 100\;$$

### 3.4. Release of BXL from SLNs

In vitro release of BXL from SLNs was assessed by the employment of 3.5 kDa cut-off dialysis tubes (Spectra/Pro, Spectrum Lab., Compton, CA, USA). One milliliter of the sample was poured into a dialysis tube, which was inserted into a beaker containing 30 mL of 50 mM TRIS buffer at pH 7.4. The solution was stirred at 200 rpm and 37 ± 0.5 °C. One milliliter of the release medium was collected at certain time points (hour by hour up to 8 h and once after 24 h) and substituted with an equal volume of fresh release medium. Samples were lyophilized and examined following the same conditions used for the entrapment efficiency and drug loading determination.

### 3.5. Differential Scanning Calorimetry

DSC studies were carried out using a Mettler Toledo STAR^e^ system (Mettler Toledo, Greifensee, Switzerland), equipped with a DSC822 calorimetric cell. Mettler TA-STAR^e^ software (version 16.00) was used to obtain and analyze data. Indium and palmitic acid (purity ≥ 99.95% and ≥99.5%, respectively; Fluka, Switzerland) were employed to calibrate the calorimeter, based on the setting of the instrument. The sensitivity was automatically chosen as the maximum possible by the calorimetric system. The reference crucible was filled with MilliQ water.

#### 3.5.1. SLNs and SLN-BXL Calorimetric Analyses

To evaluate the thermotropic behavior of the SLNs, 120 μL of each sample was hermetically sealed in a 160-μL aluminum DSC pan and was submitted to heating and cooling scans under N2 flow (70 mL/min) as follows: a heating scan from 5 to 80 °C, at 2 °C/min, and a cooling scan from 80 to 5 °C, at 4 °C/min, at least three times to confirm the reproducibility of data.

#### 3.5.2. MLV-SLNs Interaction Analysis

A total of 30 µL of multilamellar vesicles (MLVs) and 90 µL of either SLNs or BXL-loaded SLNs were dispensed and combined within a hermetically sealed DSC aluminum crucible, totaling 160 µL in volume. The system was then subjected to calorimetric analysis under a continuous flow of N2 (70 mL/min), following a prescribed protocol: (1) a heating scan from 5 to 80 °C at a rate of 2 °C/min, (2) a subsequent cooling scan from 80 to 37 °C at a rate of 4 °C/min, (3) maintaining an isothermal period of one hour at 37 °C, and (4) concluding with a cooling scan from 37 to 5 °C at a rate of 4 °C/min. This series of steps was iterated eight times for consistency and reliability.

### 3.6. Cell Cultures

HBMECs were cultured in ECM containing 5% FBS and 1% P/S solution. Cells were used for experimental procedures at approximately 70% confluence and ranged from passage P1 to P7. All the treatments were carried out in medium containing 2.5% FBS.

### 3.7. Cell Viability

Cell viability was determined using CCK-8 following the manufacturer’s instructions. Cells were treated with SLNs and SLN-BXL at 800×, 400×, 200×, 100×, 50×, and 25× dilutions, and BXL at the dilution corresponding concentrations: 3.125 μM, 6.25 μM, 12.5 μM, 25 μM, 50 μM, 100 μM. At the 24 h and 48 h time points, 10 μL of CCK-8 reagent was added to each well, followed by 2 h incubation at 37 °C, and absorbance measurement at 450 nm.

### 3.8. Wound Healing Assay

The wound-healing assay was performed to evaluate the migration ability of the HBMECs treated with 200× SLNs, 200× SLN-BXL, and 12.5 μM BXL in a medium supplemented with VEGF-A at 40 ng/mL concentration. First, 15 × 10^4^ HBMECs were seeded onto a 6-well plate. A cell-free gap was created on the confluent cell monolayers and monitored using a phase-contrast microscope after 24 h and 48 h. Cell migration was calculated as a percentage of wound closure at each time point (0 h, 24 h, and 48 h) using ImageJ software (Version 1.52a, NIH, Bethesda, MD, USA). 

### 3.9. Tube Formation

In vitro formation of capillary-like structures was studied in the Matrigel Basement Membrane Matrix system in accordance with the manufacturer’s instructions. Cells (1.5 × 10^4^ cells per well) were seeded for the following experimental conditions: CTRL (control), VEGF-A, VEGF + SLNs, VEGF + SLN-BXL, VEGF + BXL. VEGF (40 ng/mL) was added to the HBMEC medium, followed by a 6h incubation with the treatments. Cell organization onto Matrigel was observed using a phase-contrast microscope with a 10×/0.22 objective. Each experimental condition was run in triplicate. Pictures from randomly selected fields were processed using ImageJ software. ImageJ’s Angiogenesis Analyzer was used to quantify total master segment length, master junctions, total isolated branch length, and isolated segments.

### 3.10. Prostaglandin E2 (PGE2) Release

Supernatants of 24 h-incubated HBMECs with VEGF in the presence of SLNs, SLN-BXL, or BXL were collected, and aliquots were used for PGE2 quantification, using commercially available kits, following the manufacturer’s instructions (PGE2 by kit from Cayman Chemicals Co., Ann Arbor, MI, USA). For PGE2, the detection range was 7.8–1000 pg/mL. Each sample was analyzed in triplicate.

### 3.11. Interleukin-8 (IL-8) Release

Supernatants of 24 h-incubated HBMECs with VEGF in the presence of SLNs, SLN-BXL, or BXL were collected, and aliquots were used for IL-8 quantification, using commercially available kits, following the manufacturer’s instructions (IL-8 by kit from Cayman Chemicals Co., Ann Arbor, MI, USA). For IL-8, the detection range was 7.8–1000 pg/mL. Each sample was analyzed in triplicate.

## 4. Conclusions

The present research highlights the impact of the usefulness of solid lipid nanoparticles on improving the bioefficacy of a benzo[k,l]xanthene derived from natural caffeic acid phenethyl ester, CAPE-BXL. The SLNs were successfully prepared combining phase inversion temperature (PIT) and ultrasonication techniques. The SLNs were characterized for different aspects: mean particle size, polidispersity index, Z-average, entrapment efficiency, thermotropic behavior, and interaction with a model of biomembrane, and they effectively passed all these aspects. Cell viability, would healing, and tube formation assays on human brain microvascular endothelial cells were performed. Prostaglandin E2 and Interleukin-8 secretion from human brain microvascular endothelial cells was evaluated.

We considered the response of the cells constituting the human cerebral microcirculation to the tested substances, particularly to SLN-BXL, relevant in an in vitro context that mimics angiogenic sprouting. Further studies will be needed to demonstrate whether SNL, BXL, and their combination exert their effects by interacting with receptors on the cell surface, or whether they can cross the plasma membrane to modulate migration, tubulogenesis, and inflammatory production of PGE2 and IL-8 induced by VEGF in HBMECs.

In conclusion, the results suggest that this approach provides new insight into the evaluation of the efficacy of CAPE-BXL encapsulated in SLNs in disrupting angiogenesis and attenuating the inflammatory microenvironment.

## Data Availability

All data are available on request from the corresponding author.

## Supplementary Materials

The following supporting information can be downloaded at https://www.mdpi.com/article/10.3390/molecules29133103/s1: Figure S1. SEM images of (A) SLN and (B) SLN-BXL.
