# Supplementary material for: Solid Lipid Nanoparticles Encapsulating a Benzoxanthene Derivative in a Model of the Human Blood–Brain Barrier: Modulation of Angiogenic Parameters and Inflammation in Vascular Endothelial Growth Factor-Stimulated Angiogenesis
Source: Molecules. 2024 Jun 28;29(13):3103. doi: 10.3390/molecules29133103 (PMC11243179; doi:10.3390/molecules29133103)
Supplement: Supplementary file 1 [file molecules-29-03103-s001.zip › molecules-3023551-supplementary.pdf]

molecules

MDPI

Supplementary Materials

Solid Lipid Nanoparticles Encapsulating a Benzoxanthene Derivative in a Model of the Human Blood–Brain Barrier: Modulation of Angiogenic Parameters and Inflammation in Vascular Endothelial Growth Factor-Stimulated Angiogenesis

Giuliana Greco [1], Aleksandra Agafonova [1], Alessia Cosentino [1], Nunzio Cardullo [2], Vera Muccilli [2], Carmelo Pu-glia [3,4], Carmelina Daniela Anfuso [1,*], Maria Grazia Sarpietro [3,4,*] and Gabriella Lupo [1]

1 Department of Biomedical and Biotechnological Sciences, School of Medicine, University of Catania, 95123 Catania, Italy
2 Department of Chemical Sciences, University of Catania, 95125 Catania, Italy
3 Department of Drug and Health Sciences, University of Catania, 95125 Catania, Italy
4 NANOMED-Research Center on Nanomedicine and Pharmaceutical Nanotechnology, University of Catania, 95125 Catania, Italy
* Correspondence: daniela.anfuso@unict.it (C.D.A.); mg.sarpietro@unict.it (M.G.S.)
† These authors contributed equally to this work.

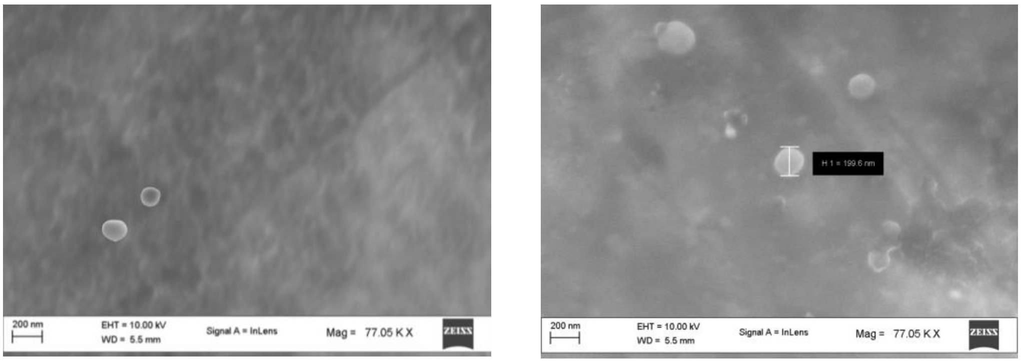

A B

Figure S1. SEM images of (A) SLN and (B) SLN-BXL.
